# Differences in Light Interception in Grass Monocultures Predict Short-Term Competitive Outcomes under Productive Conditions

**DOI:** 10.1371/journal.pone.0000499

**Published:** 2007-06-13

**Authors:** Eva Vojtech, Lindsay A. Turnbull, Andy Hector

**Affiliations:** Institute of Environmental Sciences, University of Zurich, Zurich, Switzerland; University of Sheffield, United Kingdom

## Abstract

Due to its inherent asymmetry, competition for light is thought to cause loss of diversity from eutrophied systems. However, most of the work on this topic in grasslands has been phenomenological and has not measured light directly. We present the results of one of the few mechanistic experiments investigating the outcome of short-term competition using measurements of light interception from monocultures of five perennial grass species grown under fertilized and irrigated conditions. We found that the level of incident light intercepted by each species in monoculture, a direct measure of resource-reduction ability, was an excellent predictor of the relative competitive effect in pairwise mixtures. Competition for light was asymmetric in relation to differences in light intercepting ability. Our results are consistent with the idea that when light is a limiting resource, competition between species for this resource can be asymmetric, contributing to high dominance and low diversity.

## Introduction

One of the mostly widely observed results of global change is that in many different types of ecosystems eutrophication leads to diversity loss [1]–[3]. In eutrophied terrestrial plant communities, such as many European grasslands, competition for light is thought to be a mechanism for this diversity loss [4]–[7] and the asymmetric nature of this competition can lead to an outcome which supports only low plant diversity [8]–[10]. But the details of exactly how this loss of diversity comes about are not well understood, because most studies that have been conducted so far were phenomenological.

The best developed mechanistic theory of resource-competition is Tilman's *R* ^*^ [7], [11]. If species in a system are limited by a single resource, the species that can reduce this resource to the lowest equilibrial level (*R* ^*^) is the best competitor and should displace other species. However, in terrestrial plant communities *R* ^*^ theory has only been applied to belowground resources, specifically soil nitrogen, and almost exclusively at a single site [12], [13], namely the well-known nitrogen-limited prairie at Cedar Creek, Minnesota, USA. Studies there support the ability of *R* ^*^ to predict species' relative abundances and the outcome of competition during secondary succession in old fields [13]–[17].

Huisman&Weissing [18] and Huisman *et al.* [19] are some of the few researchers to apply the *R* ^*^ approach to light. They performed competition experiments with phytoplankton in continuous, well-mixed cultures that were nutrient-rich and light-limited. They found that the critical light intensity at the bottom of a water column in monoculture (I^*^_out_), was a good predictor of competitive outcomes in species mixtures: the species with the lowest I^*^_out_ was the strongest competitor and displaced all other species. However, their cultures of phytoplankton were constantly mixed to prevent the organisms from forming layers, meaning that I^*^_out_ was directly analogous to *R* ^*^.

By contrast, in terrestrial systems, where plants establish three-dimensional canopies, species in the uppermost layer can pre-empt light and shade those beneath. A small advantage in height therefore allows much more of the light to be intercepted, conferring a disproportionately large competitive advantage. This mode of competition, which is disproportionate to some measure of size, is called relative-size asymmetric [20]–[22]. In contrast, when competition is relative-size symmetric, plants obtain a share of the resource proportionate to their size, as is often assumed to be the case when competition is for soil nutrients. With symmetric competition, growth of all plants is slowed down, whereas asymmetric competition acts to increase the variation in relative growth rates as smaller plants suffer more and therefore to exaggerate relative size differences [20]–[22]. Thus, when competition is for light, the outcome of the interaction should be quickly seen. In addition, resource utilization patterns (such as the percentage of incident light intercepted) measured during the growing season should be good mechanistic predictors of competitive outcomes. However, though some studies have shown that under conditions where competition for light is assumed to be important, competition is relative-size asymmetric [21], [23], few have included estimates of actual light interception in terrestrial habitats [21].

Here we describe a competition experiment with five perennial grass species found in European fertile meadows which were selected to differ in height (and therefore their ability to compete for light). We test the hypothesis that under productive conditions there is strong asymmetric competition for light and that the relative ability of species to intercept light predicts the outcome of competition. Although we cannot identify light as the only limiting resource, we show that this resource-based approach using light interception levels in monoculture (a measure of resource reduction) predicted short-term competitive outcomes, and confirm that competition for light was asymmetric.

## Results

### Outcome of Short-Term Competition for Light

**Figure 1:**
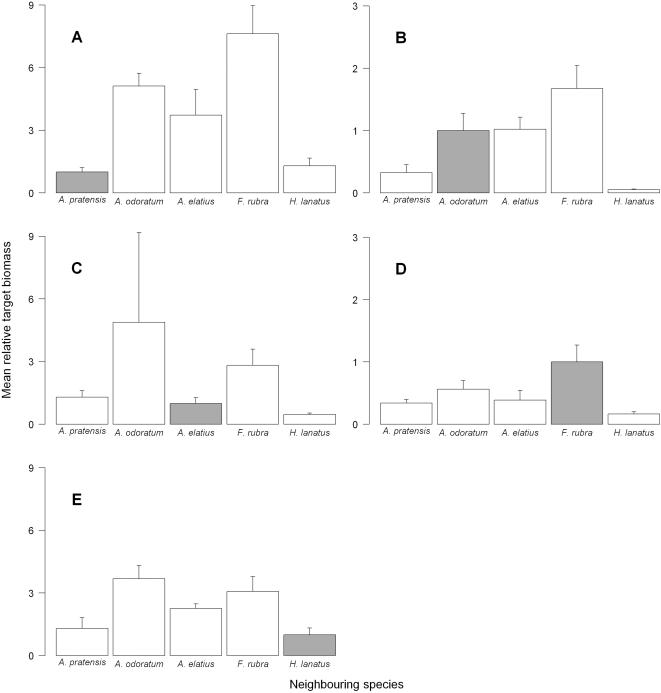
Mean relative target biomass of all species in the control treatment. Mean target biomass (±SE) of each species in all pairwise combinations, standardized by the target biomass with conspecific neighbours. Grey bars indicate target biomass with conspecific neighbours, white bars target biomass with their respective interspecific neighbours. Target species are: (A) *A. pratensis*, (B) *A. odoratum,* (C) *A. elatius*, (D) *F. rubra* and (E) *H. lanatus.*

The observed competitive hierarchies averaged over all target-neighbour species combinations were: *H. lanatus*>*A. pratensis*>>*A. elatius*>>>*A. odoratum*>*F. rubra* (where ”>” means “had an overall higher relative competitive effect than”). Our species had highly unequal abilities to suppress target plant growth of the other species. Target plant biomass of *F. rubra* and *A. odoratum* decreased strongly when they were surrounded by the taller *A. pratensis* and *H. lanatus*, in comparison to their biomass when growing surrounded by conspecifics (Fig. 1). In contrast, target biomass of *A. pratensis* and *H. lanatus* increased strongly when they were surrounded by the shorter *F. rubra* and *A. odoratum* in comparison to their biomass when they were surrounded by conspecifics (Fig. 1).

**Figure 2:**
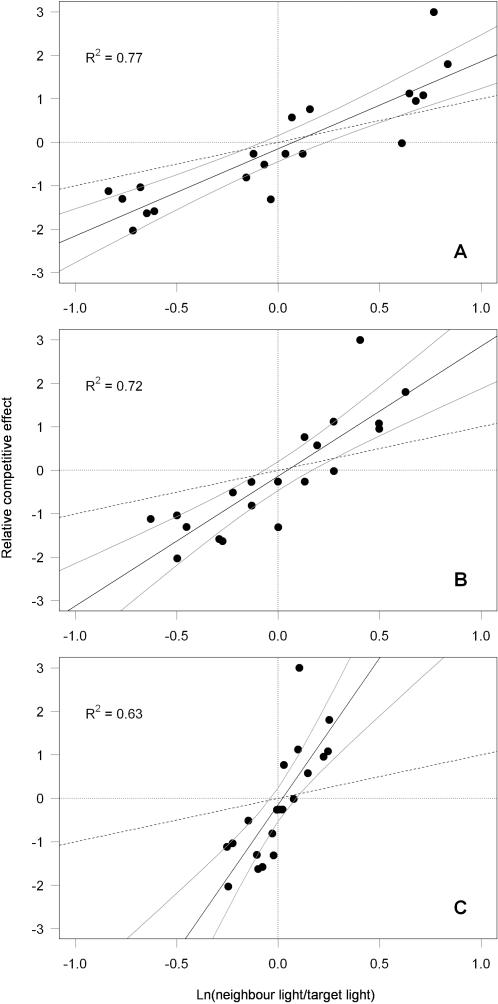
Relationship between the relative competitive effect and relative differences in light interception. Linear regression slopes and 95% confidence intervals for the relationships between the relative competitive effect (*RCE_ij_*) and the log ratio of neighbour/target light interception levels (A) 10 weeks, (B) 15 weeks and (C) 18 weeks after sowing. The black dashed line is the expected regression line with perfect symmetry which has a slope of one and an intercept of zero.

The relative competitive effect was significantly positively related to relative differences in light interception in monoculture–measured 10, 15 and 18 weeks after sowing (linear regression with 95% confidence intervals, Fig. 2). This shows that species intercepting a greater percentage of incident light–and thus reducing the light available to species with lower canopies–had a competitive advantage. The later in the season the light interception measurement was taken, the smaller the relative differences in light interception levels between species and the less variation in the relative competitive effect was explained (Fig. 2).

**Figure 3:**
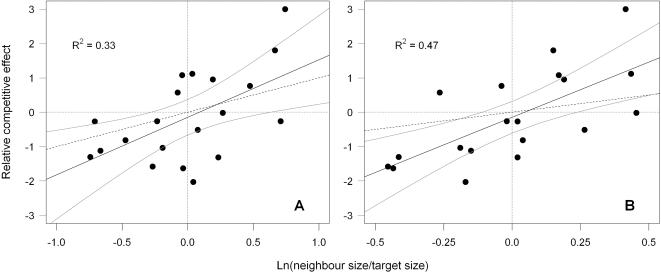
Relationship between the relative competitive effect and relative differences in sizes. Linear regression slopes and 95% confidence intervals for the relationships between the relative competitive effect (*RCE_ij_*) and the log ratio of neighbour/target values for (A) monoculture biomass, (B) maximum monoculture canopy height. The black dashed line is as in Fig. 2.

The relative competitive effect was also significantly positively related to relative differences in species' sizes (linear regression on monoculture biomass with 95% CIs: Fig. 3A; maximum monoculture canopy height: Fig. 3B).

### Competitive Asymmetry

We tested for symmetry by checking whether the 95% confidence intervals for the linear regression slopes contained the predicted value of +1. All slopes were greater than +1 with no confidence interval containing that value: 10 weeks (Fig. 2A, slope = 2.0 (1.46–2.54)), 15 weeks (Fig. 2B, slope = 3.0 (2.07–3.90)) and 18 weeks (Fig. 2C, slope = 6.7 (4.19–9.29)). The relationship was more asymmetric when the values from later measurements of light interception levels were used, due to the decreasing differences in the light interception levels between species as they approached maximum canopy height.

Tests for relative size-asymmetry depended on the measurement of size used. The confidence intervals for the relationship between the relative competitive effect and relative difference in aboveground monoculture biomass did contain +1, consistent with relative size-symmetric competition (Fig. 3A, slope = 1.7 (0.49–2.86)). By contrast, the confidence intervals for the relationship between the relative competitive effect and relative difference in maximum monoculture canopy height did not contain +1, indicating an asymmetric advantage (Fig. 3B, slope = 3.2 (1.50–4.91)). Taken together, this implies that greater maximum canopy height and increased ability to intercept incident light confer a disproportionately large competitive advantage. This confirms that competition for light was asymmetric under the productive conditions of our experiment as predicted.

### Manipulation of Competition for Light

**Figure 4:**
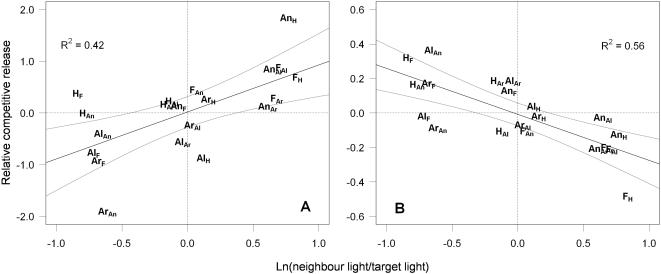
Relationship between the relative competitive release and relative differences in light interception. Linear regression slopes and 95% confidence intervals for the relationships between the relative competitive release (*RCR_ij_*) based on (A) target plant biomass and (B) height and the log ratios of neighbour/target light interception levels 10 weeks after the sowing. Letters denote the target plant species; subscripts denote the corresponding surrounding species of each respective species pair, as given in Fig. 1.

As the light interception level measured 10 weeks after sowing was the best single predictor for the relative competitive effect, we used this variable to investigate the relative competitive release (i.e. the response of target plant biomass and height to netting away neighbours, corrected for the performance of target individuals with conspecific neighbours). As expected there was a significant positive relationship between the relative competitive release based on target plant biomass and the difference in light interception levels measured in monoculture (Fig. 4A; linear regression slope = 0.9 (0.38–1.44)). This shows that the magnitude to which species were released from competition depended on the relative light interception capabilities between each species pair. By contrast, the slope of the relationship between the relative competitive release based on target plant height and differences in light interception levels measured in monoculture was negative (Fig. 4B; slope = −0.3 (−0.15– −0.39)). Thus, the greatest *increase* in biomass was seen when the best light competitor (species intercepting the most light) was netted away from the poorest light competitor (the species intercepting least light) and this was accompanied by the greatest *decrease* in height. The change in height presumably reflects a plastic response to the reduction of competition which removes the need to grow tall.

## Discussion

The aim of this experiment was to test how well the differences in the light-intercepting abilities of our deliberately selected species could mechanistically explain the short-term outcome of competition under conditions where there was strong competition for light. While we cannot exclude additional competition for other resources, we found that there was strong asymmetric competition for light and that short-term competitive outcomes could be well predicted by differences in the percentage of intercepted incident light of each species in monoculture. The species that intercepted the greatest percentage of light had the greatest relative competitive effect (Fig. 2). Species differences in light interception also determined the relative response of species to the netting treatment, in which aboveground competition was reduced (Fig. 4), and confirmed that our species were highly unequal competitors. Compared with the full competition treatment, target plant biomass of *A. odoratum* and *F. rubra* (poorest light competitors) increased by an average of 47% in the netting treatment when either *A. pratensis* or *H. lanatus* (best light competitors) were neighbours. On the other hand, *A. pratensis* and *H. lanatus* showed very little change in biomass following netting when *A. odoratum* or *F. rubra* were neighbours.

Although some studies have shown that under conditions where competition for light is important, competition is relative-size asymmetric [21], [23], few have included estimates of the actual light interception in terrestrial habitats [21]. By investigating the slopes of the relationship between the relative competitive effect and relative differences in light interception, we were able to show that competition was asymmetric in regard to light interception (Fig. 2). Additionally, we have also found relative-size asymmetry in the relationship between the relative competitive effect and relative differences in maximum canopy height (Fig. 3B). This implies, that a species with greater maximum canopy height which therefore intercepted more incident light, would have a disproportionate competitive advantage. Our study agrees with the following two studies that also included estimates of actual light interception. A study of intraspecific competition between birch seedlings showed that the tallest individuals within a population intercept the majority of light at the expense of shorter individuals [24]. Schwinning [25] found a positive slope in the relationship between the log ratio of light interception differences and the differences in biomass of two individuals in the high density treatment of millet plants, a positive slope in the relationship between light interception per unit leaf area and dry shoot biomass and concluded that at high density, competition for light can be asymmetric. More recently, Dybzinski&Tilman [26] have demonstrated that *I* ^*^ can be used to successfully predict longer-term (11 years) competitive exclusion in a nitrogen gradient at Cedar Creek.

According to the *R* ^*^ theory for soil nutrients, equilibrial resource levels are required to explain competitive outcomes, because growth of all competitors is equally limited by the lack of resource and the strength of a competitor shows in its ability to persist at a resource level that is lower than that of other competitors. However, asymmetric competition increases relative size differences between species [21], [22] and enables species intercepting more of the incident light to maintain their initial dominant position during the whole growing season. Under such circumstances, dominance and even competitive exclusion can develop very quickly. This implies that measurements of intercepted light taken at early stages of vegetation growth should be good predictors of competitive outcomes. All of our three light measurements (10, 15 and 18 weeks after the sowing of the experiment) gave good qualitative predictions of the relative competitive effect. However, in accordance with a recent study by Violle *et al.* [27], differences in light interception in monocultures at the earliest measurement (after 10 weeks) best explained competitive outcomes at harvest (18 weeks). Both studies therefore agree that instantaneous measurements of light interception can be very useful predictors, as long as they are obtained during a critical time when light becomes limiting for plant growth [27].

Because competition for light acts essentially instantaneously on quickly developing communities such as grasslands, short-term experiments can give valuable insight into underlying mechanisms. In the long-term, other factors and trade-offs might of course modify the outcomes of competition and reduce the predictive power of intercepted light in our system. For example, founder effects may play an important role when competition is for light [28]–[30]. Litter accumulation over long time intervals can also lead to reduced light intensities and have thus important effects on seedling recruitment and plant biodiversity [31], [32]. However, for the reasons outlined above, we expect little scope for transient effects to occur when competition for light is as considerable as in our experiment, and thus also little potential for a mis-match between short- and long-term competitive outcomes. Our study could not test for limitation by all potential resources and so we cannot exclude an additional role of other forms of competition. Nevertheless, we have shown that under productive conditions the short-term outcome of competition in our experiment could be well predicted from a resource-based predictor: light interception (resource reduction) in monoculture. Our study is therefore consistent with competition for light as an important component of mechanisms of competitive exclusions in productive and eutrophied grasslands.

## Materials and Methods

### Experimental Design

The competition experiment reported here is part of a wider project about light competition and partitioning in grasslands which uses a model system of five perennial grass species (Poaceae) [cf. 17] parsimoniously selected from those found in European fertile meadows to differ in their canopy heights and light competition abilities. The species are: *Alopecurus pratensis* L., *Anthoxanthum odoratum* L., *Arrhenatherum elatius* (L.) P. Beauv. ex J. Presl&C. Presl, *Festuca rubra* ssp. *commutata* Gaud. ( = *Festuca nigrescens* Lam.), *Holcus lanatus* L. [33]. The experiment was conducted in the experimental garden of the Institute of Environmental Sciences, Zurich (47°23′N, 8°33′E, and 546 m height a.s.l.).

One central target plant was grown surrounded by a ring of neighbours of each of the species including itself (i.e. in all possible intraspecific and interspecific pairwise combinations), in plots defined by PVC rings of 30 cm diameter and filled to a depth of 15 cm with a highly-fertile soil. The neighbour species were sown at a density of 1000 seeds m^−2^ (corrected based on the results of prior germination trials). Aboveground competition between the neighbours and the target species was successfully reduced by tying back the neighbouring vegetation with fine tree netting. Plants were watered with an automatic irrigation system on a daily basis. The 25 target-neighbour species combinations crossed with the control and reduced aboveground competition treatments produced 50 combinations which were repeated five times in a randomised block design giving a total sample size of 250 plots. The neighbouring species were sown in April. Target seedlings were transplanted to the experimental plots one month later. At this time the targets had approximately the same size as the neighbour plants. At the end of August (approximately 18 weeks after the sowing), aboveground parts of target plants were harvested, dried at 80°C and weighed.

### Analysis of Competition and Competitive Asymmetry

For the control (full competition) treatments, we calculated the relative competitive effect of each neighbour species on each target species and related these competitive effects to differences in light-depletion levels and to species' sizes [cf. 34], [35]. The relative competitive effect (*RCE_ij_*) of each neighbour species, *j*, on each target species, *i*, was calculated as the log ratio: (1)
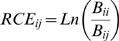
where *B_ii_* is the biomass of target species *i* surrounded by conspecific neighbours, and *B_ij_* is the biomass of target species *i* surrounded by neighbours of species *j*. A positive value of the relative competitive effect means that the target biomass was lower when growing with species *j* neighbours than with conspecific neighbours, i.e. neighbours of species *j* have a stronger negative effect on the target biomass than conspecific neighbours, and vice versa. Competitive hierarchies were established by averaging over the ability of species to competitively suppress the other four species.

Light interception in monocultures (measured during the first growth season 10, 15 and 18 weeks after the sowing), and measures of species' sizes (aboveground monoculture biomass and maximum canopy height in monoculture, measured from ground to the highest leaf) were obtained from a companion experiment started in spring 2004. It consists of 80 1 m^2^ plots where the same five grass species as in the competition experiment described here are grown on highly fertile soil in monocultures, pairwise mixtures and the five-species mix. Light levels were measured above and below monoculture canopies (approximately at ground level) with a photosynthetically active radiation probe (SunScan System-SS1, Delta-T Devices Ltd, Cambridge, UK) and the percentage of incident light intercepted in each canopy was calculated. The relative difference in light intercepted in monoculture between species *i* and *j* was calculated using the log ratio: (2)
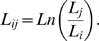
A positive value of *L_ij_* means that the neighbouring species *j* intercepted more light in monoculture than the target species *i* and vice versa. Similarly, the relative difference in species' sizes was calculated as S*_ij_*, the log ratio of *S_j_* (aboveground monoculture biomass or maximum monoculture canopy height of the neighbouring species) and *S_i_* (aboveground monoculture biomass or maximum monoculture canopy height of the target species).

We can quantify the relationship between the relative competitive effect and relative differences in light interception and test for symmetry. If competition is symmetric and *L_ij_* = N we expect *RCE_ij_* = N. However, under asymmetric competition, when *L_ij_* = N we expect *RCE_ij_*>N, i.e. the difference in trait values has conferred a disproportionate competitive advantage. Thus, plotting *RCE_ij_* against L*_ij_* should reveal a slope of +1 if competition is symmetric, or>+1 if competition is asymmetric. We chose the percentage of incident light intercepted in monoculture (*L* = 100-*I* ^*^) instead of the absolute light level below the monoculture (*I* ^*^) for two reasons (see Text S1).

### Manipulation of Competition for Light

The netting treatment was used to confirm that there was competition for light. When there is competition for light, we expect a poor light competitor surrounded by a good light competitor to respond to the netting with a large increase of biomass, because in this case tying back the neighbour should reduce shading. In the opposite case, when a good light competitor is surrounded by a poor light competitor, we would expect no or only a small increase of biomass, because there is little shading. Thus, the magnitude of release from competition due to the netting should depend on the relative light interception capabilities between each species pair. To assess the response of target plant biomass to the reduction of aboveground competition we calculated the relative competitive release (*RCR_ij_*) which is the log ratio of the inverse of the relative competitive effects in the control and netting treatments: (3)
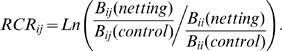
The relative competitive release is positive when the target biomass (B) of species *i increases* more when neighbours of species *j* are netted away than when conspecific neighbours are netted away. This occurs when the relative competitive effect of species *j* on target species *i* is large and positive and vice versa.

In addition we also calculated the relative competitive release using target plant height instead of biomass. Plants can respond plastically to shading by increasing their height (but not their biomass) in an attempt to escape shading. Thus, we expect to find that target plants experiencing substantial shading by a neighbouring species *j* will decrease in height in the netting treatment, whereas target plants experiencing no or only slight shading by a neighbouring species *j* should show no decrease in height. In this case, the relative competitive release calculated using height rather than biomass is expected to be negative when the relative competitive effect of an interspecific neighbour is large and positive; that is when the target plant height *decreases* more when species *j* neighbours are netted away than when conspecific neighbours are netted away and vice versa.

## Supporting Information

Text S1 Additional information on the analysis of competition. Reasons why we chose the percentage of incident light intercepted in monoculture instead of the absolute light level below the monoculture.
(0.03 MB DOC)
