# Supplementary material for: Differences in Light Interception in Grass Monocultures Predict Short-Term Competitive Outcomes under Productive Conditions
Source: PLoS One. 2007 Jun 13;2(6):e499. doi: 10.1371/journal.pone.0000499 (PMC1885551; doi:10.1371/journal.pone.0000499)
Supplement: Text S1 — Additional information on the analysis of competition. Reasons why we chose the percentage of incident light intercepted in monoculture instead of the absolute light level below the monoculture. (0.03 MB DOC) [file pone.0000499.s001.doc]

**Text S1: Additional information on the analysis of competition**

Reasons why we chose the percentage of incident light intercepted in monoculture instead of the absolute light level below the monoculture:

We chose the percentage of incident light intercepted in monoculture (*L* = 100-) instead of the absolute light level below the monoculture () for two reasons. First, because intercepted light can be directly converted into growth by the plant and is no longer available to plant competitors, whereas provides information about the level of unused resource. Second, using the percentage of intercepted light also has advantages for investigating symmetry. Consider two species where one intercepts 90% of the incoming light while the other intercepts 45% (i.e. the first intercepts twice as much light). Then using Eqn 2 from the text, *L_ij_* = *Ln*(90/45) = *Ln*(2) = 0.69. If competition is symmetric with regard to light interception, we expect *RCE_ij_* to be also *Ln*(90/45), because when species *j* intercepts twice as much light as species *i*, it should also have twice the competitive effect. In contrast, using directly gives a value of *Ln*(10/55) = -1.7. The same test can be performed to test for relative-size symmetry in the relationship between *RCE_ij_* and *S_ij_*.
